# Healthy Dietary Changes in Midlife Are Associated with Reduced Dementia Risk Later in Life

**DOI:** 10.3390/nu10111649

**Published:** 2018-11-03

**Authors:** Shireen Sindi, Ingemar Kåreholt, Marjo Eskelinen, Babak Hooshmand, Jenni Lehtisalo, Hilkka Soininen, Tiia Ngandu, Miia Kivipelto

**Affiliations:** 1 Division of Clinical Geriatrics, Center for Alzheimer Research, Karolinska Institutet and Karolinska University Hospital, SE-17176 Stockholm, Sweden; babak.hooshmand@ki.se (B.H.); Tiia.ngandu@thl.fi (T.N.); Miia.kivipelto@ki.se (M.K.); 2 Neuroepidemiology and Ageing Research Unit, School of Public Health, Imperial College London, London SW72BX, UK; 3 Aging Research Center, Karolinska Institutet and Stockholm University, SE-10691 Stockholm, Sweden; Ingemar.kareholt@ki.se; 4 Institute of Gerontology, School of Health and Welfare, Aging Research Network—Jönköping (ARN-J), Jönköping University, SE-55111 Jönköping, Sweden; 5 Institute of Clinical Medicine, Department of Neurology, University of Eastern Finland, FI-70211 Kuopio, Finland; marjo.Eskelinen@uef.fi (M.E.); Hilkka.Soininen@uef.fi (H.S.); 6 Chronic Disease Prevention Unit, National Institute for Health and Welfare, FI-00271 Helsinki, Finland; jenni.lehtisalo@thl.fi; 7 Neurocenter, Department of Neurology, Kuopio University Hospital, FI-70029 Kuopio, Finland; 8 Theme Aging, Karolinska University Hospital, SE-17176 Stockholm, Sweden; 9 Research and Development Unit, Stockholms Sjukhem, 11219 Stockholm, Sweden

**Keywords:** diet, dietary change, midlife protective factors, dementia, public health

## Abstract

Diet is an important modifiable lifestyle factor related to dementia risk. Yet, the role of midlife dietary changes is unclear. The goal is to investigate whether midlife healthy dietary changes are associated with late-life dementia risk. Data were collected within the Cardiovascular Risk Factors, Aging, and Dementia (CAIDE) population-based cohort study (*n* = 2000) (mean baseline age = 56 years). Participants returned for two late-life re-examinations (mean age = 70 and 78 years). Self-reported midlife diet was measured in a sub-sample (*n* = 341) (mean total follow-up = 16.8 years). Changes in specific dietary components (fats, vegetables, sugar, salt) were measured in midlife. Dementia diagnoses were ascertained with detailed examinations. Analyses adjusted for potential confounders. Total midlife healthy dietary changes (improving quality of fats, increasing vegetables, decreasing sugar and salt) were associated with a reduced risk of dementia (fully adjusted odds ratio (OR) 0.41, 95% confidence interval (CI) = 0.20–0.85). In contrast, when each factor was assessed individually, associations were not significant. This study is the first to show that beneficial midlife dietary changes are associated with a reduced dementia risk later in life. The results highlight the importance of targeting dietary patterns, where various food items may have synergistic effects.

## 1. Introduction

In 2015, it was estimated that 46.8 million people were living with dementia. This number is projected to increase to 75 million in 2030, and 131.5 million in 2050 [1]. In the absence of disease-modifying drugs, prevention has been highlighted as a public health priority. At least a third of dementia cases may be preventable through lifestyle modifications [2,3]. As the processes leading to dementia (including brain pathology) may start 20 years prior to dementia onset [4,5], it is crucial to start healthy lifestyle modifications at earlier stages in life (e.g., in midlife or earlier).

Diet is an important modifiable lifestyle factor related to dementia risk. In the past two decades, numerous studies have highlighted the role of diet as a potential protective lifestyle factor. Many studies have focused on dietary patterns such as the Mediterranean diet, Western vs. traditional diets, showing that higher adherence to healthy diets is associated with a reduced risk of dementia (for recent systematic reviews and meta-analyses see [6,7,8,9]). Collectively, these studies show that diets rich in antioxidants (e.g., from fruits, vegetables, coffee), healthy fats (e.g., omega-3 fatty acid from fish, nuts, olive oil), with low‒moderate red meat consumption, and low consumption of refined sugars, carbohydrates and unhealthy fats may protect against dementia [6,7,8,9].

Other studies have examined specific dietary factors such as various vitamins (e.g., vitamins C, D, E) or multivitamins, omega-3 fatty acids and other polyunsaturated fatty acids, flavonoids, folate or other macronutrients (e.g., cholesterol, total fat, saturated fat or trans fats), with mixed results [6,7,8,9]. It has also been highlighted that diet may interact with genetic risk factors for dementia, namely the Apolipoprotein E4 (APOE ε4) allele [10,11,12], which is important to take into consideration when investigating the associations between diet and dementia.

To date, many studies have focused on the associations between individuals’ current dietary patterns (e.g., the Mediterranean diet) or their consumption of specific macronutrients and the associations with dementia risk. However, it is unknown whether a healthy change in diet (e.g., increasing the intake of beneficial food items while decreasing the intake of unhealthy food items) is associated with later risk of dementia.

The aim of this study was to investigate whether healthy dietary changes in midlife are associated with dementia risk later in life, using a population-based cohort study (total follow-up: 22 years).

## 2. Materials and Methods

### 2.1. The CAIDE Study

The Cardiovascular Risk Factors, Aging and Dementia (CAIDE) study participants were first examined at midlife (baseline) in the North Karelia Project and the FINMONICA population-based studies in Finland. Baseline assessments were carried out in one of the following years: 1972, 1977, 1982, or 1987 [13]. Baseline participation rates ranged between 82% and 90%. In 1998, a random sample of 2000 survivors (living in the cities of Kuopio and Joensuu), aged 65–79, were invited for the first re-examination. A total of 1449 individuals (72.5%) participated and 1409 underwent the cognitive assessments. The mean total follow-up time was 21 years. Participants returned for the second re-examination between 2005 and 2008 (mean follow-up: eight years, standard deviation (SD) = 1.0) after first re-examination). In 2005, 1426 (of the original sample of 2000) were alive and living in the same city/region. Upon invitation, 909 (63.7%) of these agreed to participate and 852 underwent the cognitive assessments.

### 2.2. Study Population

In total, 1511 individuals participated in at least one re-examination, and 750 participated in both. Information on changes in diets and a questionnaire on diet were available from 341 participants assessed in 1982 and 1987 (from a total of 634 who had information on dementia diagnosis). Among this sub-sample of 341 participants, 194 participants participated in both re-examinations.

In order to maximize the sample size, all subjects with a dementia diagnosis in at least one re-examination were included in the analyses. Data were organized in what is referred to as long format, which offers 535 observations. In this format, participants with dementia diagnoses at both the first and second re-examinations are included twice in the dataset, with one observation for the first re-examination and one observation for the second re-examination. The few participants who had dementia at the first re-examination and were still alive at the second re-examination were excluded from the second re-examination. This means that each participant with dementia was only included once.

### 2.3. Ethical Considerations

The CAIDE study was approved by local ethics committees in Kuopio and Joensuu. All participants provided written informed consent.

### 2.4. Healthy Dietary Changes

Self-reported dietary changes were measured at midlife with the following question:

Have you over the last year changed your eating habits based on health considerations? Answers were provided for the following statements: (1) Have modified the quality of fats during the last year; (2) Have increased the consumption of vegetables during the last year; (3) Have decreased the use of sugar during the last year; (4) Have decreased the use of salt during the last year. Response options were the following: 1 = No, 2 = yes. A total score for healthy dietary changes was computed by adding all four dietary change components to create a total healthy dietary change measure. The scores ranged from 4 to 8. A higher score indicated more healthy dietary changes.

### 2.5. Formation of a Healthy Diet Index

Information on diet was acquired through a questionnaire on diet that was administered in midlife. Items selected for the healthy diet index were those that correspond to the healthy dietary changes questions (described above, regarding fats, vegetables, salt and sugar). Consumption of berries/fruits was included with the consumption of vegetables, as they are similar in: (a) high levels of antioxidant properties including phytochemicals and vitamins, (b) high fiber content, (c) low calories [14].

For details on the food items included, as well as the response options, see Table 1. A healthy diet index was calculated by initially performing median splits for each item (below the median (unhealthy diet) = 0; equal and above the median (healthy diet) = 1). For example, the question “How often have you eaten vegetables or root vegetables (not potatoes) as such, grated, or as fresh salads during the past week (seven days)?’’ was dichotomized between 0.33 (1–2 days) and 0.67 (3–5 days). Therefore, participants eating vegetables on two days or fewer received a score of 0, whereas participants eating vegetables three days or more received a score of 1. In order to prevent an over-representation of specific dietary items, each dietary item was divided by the total number of items for that category and then summed. For example, since there were four items for fats, they were each divided by four before being summed, to achieve a better overall balance of food categories.

### 2.6. Diagnosis of Dementia in the CAIDE Study

Cognitive performance was assessed at both re-examinations using a three-step dementia diagnosis protocol: screening phase, clinical phase, and differential diagnostic phase. At the first re-examination (1998), participants who scored ≤24 on the Mini-Mental State Examination (MMSE) [15] at screening were referred for further examinations in the clinical phase. At the second re-examination (2005–2008), participants were referred to the clinical phase if they scored ≤24 points on MMSE, or had a decline of ≥3 points on MMSE since 1998, or if they scored <70% in delayed recall on the Consortium to Establish a Registry for Alzheimer’s Disease (CERAD) word list [16], or if the participant’s informant expressed concerns regarding his/her cognition. These criteria were added to increase sensitivity for the detection of milder cognitive impairment. The clinical phase (at both re-examinations) involved detailed neurological, neuropsychological, and cardiovascular examinations, and the differential diagnostic phase involved brain imaging (Magnetic Resonance Imaging (MRI)/Computerized Tomography (CT)), blood tests, and, if needed, cerebrospinal fluid analysis and electrocardiogram. A review board (including the physician, senior neurologist, and neuropsychologist) used all assessment information to specify the primary diagnosis. Dementia diagnosis criteria were based on the Diagnostic and Statistical Manual of Mental Disorders (DSM) [17]. Alzheimer’s disease diagnosis criteria were based on the National Institute of Neurological and Communicative Disorders and Stroke and the Alzheimer’s Disease and Related Disorders Association (NINCDS-ADRDA) [18].

### 2.7. Other Assessments

Standardized assessments and survey methods at baseline (midlife) conformed to international guidelines and the World Health Organization (WHO) (Multinational MONItoring of trends and determinants in CArdiovascular disease) MONICA protocol [19]. Re-examination surveys were similar and comparable with baseline surveys. The surveys included questionnaires on sociodemographic factors, health status, medical history, and health-related behaviors. Education was measured at baseline by asking participants about their highest level of educational attainment. Physical activity was measured with the following question: “How often do you participate in leisure-time physical activity that lasts at least 20–30 min and causes breathlessness and sweating?” Response options were 1 = daily; 2 = 2–3 times a week; 3 = once a week; 4 = 2–3 times a month; 5 = a few times a year; and 0 = not at all. To obtain an ordinal variable, “not at all” was recoded into 6. A venous blood sample was obtained for measures of biomarkers, including APOE ε4 genotype from blood leucocytes analyzed using HHaI digestion and polymerase chain reaction [20]. The Hospital Discharge Register (starting in 1967) was used to acquire information on diagnoses of respiratory and cardio/cerebrovascular conditions (chronic obstructive pulmonary disease, asthma, coronary artery disease, stroke, myocardial infarction, atrial fibrillation, cardiovascular surgery, heart failure, or diabetes).

## 3. Statistical Analyses

Analyses were performed using Stata 13.0 (Stata Corp, College Station, TX, USA). The significance level for all analyses was set at *p* < 0.05.

We performed logistical regressions for the associations between midlife healthy dietary changes and dementia later in life (at the first or second re-examinations). Results are reported as odds ratios (OR) and 95% confidence intervals (CI). All analyses were adjusted for basic confounders: age, sex, education, baseline cohort (Model 1). Subsequent analysis adjusted for healthy diet index (Model 2), in order to explore if any association is due to reported changes in diet or the actual diet. Additional analysis adjusted for APOE ε4 status (participants with one or two APOE ε4 alleles were categorized into one group), baseline cardio/cerebrovascular or respiratory conditions, baseline body mass index (BMI), and baseline self-reported physical activity (Model 3).

## 4. Results

### Population Characteristics

Table 2 presents the sociodemographic and clinical characteristics of the participants included in the analyses. Data for categorical variables are reported as proportions, while data for continuous variables are reported as means (SD). Table 3 shows the associations between midlife healthy dietary changes and late-life dementia. Total midlife healthy dietary changes (including modifying quality of fats, increasing vegetables, decreasing sugar and decreasing salt) were associated with a reduced risk of dementia (fully adjusted OR = 0.41, 95% confidence interval (CI) = 0.20–0.85). In contrast, when each of these factors was assessed individually, they were not significantly associated with dementia risk. The only exception was for decreasing sugar, which was associated with a reduced risk of dementia in the basic model 1 (adjusting for age, sex, cohort and education, OR = 0.36, 95% confidence interval (CI) = 0.13–0.97)), and model 2, which additionally adjusts for the healthy diet index (OR = 0.37, CI = 0.14–0.99). However, when model 3 additionally adjusts for cardio/cerebrovascular or respiratory conditions, BMI, APOE ε4 and physical activity, the associations between sugar reduction and dementia risk were no longer significant.

## 5. Discussion

This study showed that healthy dietary changes in midlife, characterized by modifying the quality of fats, increasing vegetable consumption, and decreasing salt and sugar consumption, are associated with a reduced risk of dementia later in life. Changes in only one of the dietary components (e.g., salt, quality of fats) was not associated with a reduced dementia risk. These findings remained significant after adjusting for several potential confounding factors.

These results support previous findings suggesting synergistic effects between various food components [21] and the risk of dementia [22]. For example, phytochemicals (e.g., from fruits and vegetables) may have synergistic effects when combined with fatty acids (e.g., from fish) [23]. Synergistic effects may also be due to the impact of absorption when different foods are combined, for example, copper‒zinc and manganese iron, or vitamin C and iron (for a review, see [21]). These results highlight the importance of targeting whole dietary patterns, instead of promoting/discouraging the consumption of single nutrients.

The results showed that decreased sugar consumption was associated with a reduced risk of dementia, even after adjusting for the healthy diet index. However, these findings were no longer significant when we adjusted for cardio/cerebrovascular or respiratory conditions, BMI, APOE ε4 and physical activity. These effects warrant further investigation, especially as it was recently shown that higher consumption of sweetened beverages was associated with markers of pre-clinical Alzheimer’s disease in a cross-sectional study [24]. Caution should also be exercised when recommending alternative artificial sweeteners, as they have been associated with an increased risk of dementia and stroke [25].

These findings are important for dementia prevention initiatives. While many dementia risk factors are important in midlife (e.g., high BMI, hypertension, physical inactivity), at-risk individuals may not be motivated to make lifestyle changes as the potential for dementia onset may be perceived as distant. These findings highlight the importance of dietary changes in midlife, and their benefits for dementia risk reduction decades later. Motivational strategies may be used so that at-risk individuals are encouraged to make healthy lifestyle changes at an earlier stage in life, as well as maintain healthy lifestyles for the following years in order to reduce their risk of dementia.

In the current study, the dietary changes may have been the result of public health campaigns in Finland in the 1970s. By the early 1970s, Finland had the highest rates worldwide of mortality from ischemic heart disease. Prevention of cardiovascular conditions among other non-communicable diseases became a priority for Finland’s national health policy [26]. The North Karelia project was launched to assess whether key risk factors (e.g., blood pressure, cholesterol, smoking) can be reduced in the population, and subsequently decrease the rates of mortality related to cardiovascular conditions. Through the involvement of non-governmental organizations, health services, various campaigns, industry, and the media, the project widely disseminated information on healthy lifestyles and how to implement behavioral changes (e.g., for healthy diets, smoking cessation, and increasing physical activity). As a result, between 1970 and 1995, the rate of coronary heart disease decreased by 73% in North Karelia, and 65% in Finland as a whole. The North Karelia project provided the world with an exemplary model of national prevention strategies, and the current study additionally shows that such lifestyle changes can have long-lasting effects on dementia risk several decades later.

Various potential mechanisms may underlie these findings. Higher adherence to diets that are rich in anti-inflammatory and antioxidant properties (e.g., the Mediterranean diet, which is similar to the Nordic diet) are associated with longer leukocyte telomere length [27,28,29,30], and reduced risk of cardiovascular diseases [31]. Dietary patterns high in consumption of red/processed meat and fried foods are associated with elevated levels of inflammatory markers (interleukin-6) and rapid cognitive decline [32]. In contrast, omega-3 fatty acids are known to have anti-inflammatory capacities [33]. Antioxidant trace elements (e.g., zinc and selenium) may offer neuroprotection against oxidative stress [34], and it has been shown that adherence to a Mediterranean diet is associated with reduced inflammation and increased antioxidant-related functions [35].

This study has several strengths, including a long follow-up duration, comprehensive examinations for dementia diagnoses, and having adjusted for important confounding factors. This study also has some limitations. The midlife measures of diet were only assessed at one time point, so it is unclear whether the reported changes were sustained throughout the year. We also could not take various types of fish fats into account when calculating the diet index, and this is important considering the potential protective role of omega-3 fatty acids against the risk of cognitive impairment and dementia [36]. The sample size did not allow us to investigate interaction effects with the APOE ε4 genetic allele. The dietary change questions are subjective, and caution is needed when interpreting the findings. It will be important to replicate our findings in samples that have collected dietary data at several time points during midlife, and have sufficient power to investigate the associations with various dementia subtypes.

In conclusion, this study shows that healthy dietary changes in midlife are associated with a reduced risk of dementia. Investments in public health initiatives to promote a healthier diet, in addition to improving other lifestyle factors (e.g., increasing physical activity), may have long-term benefits for the prevention of conditions that are more prevalent with aging.

## Figures and Tables

**Table 1:** Formation of a healthy diet index.

Food Item	Question	Response Options (Score in the Healthy Diet Index) Cutoff for Healthy Diet Change
**Vegetables/berries**	How often have you eaten vegetables or root vegetables (not potatoes) as such, grated, or as fresh salads during the past week (seven days)?	Not one (0) 1–2 days (0.33) 3–5 days (0.67) 6–7 days (1)
	How often have you eaten fresh or frozen berries or fruits during the past week?	Not one (0) 1–2 days (0.33) 3–5 days (0.67) 6–7 days (1)
**Fats**	What fat in your home is usually used for cooking?	Mostly vegetable oil (1) Mostly soft spread margarine (0.5) Mostly margarine (0.5) Mostly butter and oil mixture (0) Mostly butter (0) Food is not cooked in my home (0.5)
	What fat in your home is usually used for baking?	Mostly vegetable oil (1) Mostly soft spread margarine (0.5) Mostly margarine (0.5) Mostly butter and oil mixture (0) Mostly butter (0) I do not bake at home (0.5)
	If you drink milk, do you usually use:	Ordinary cow’s milk (≈3.8% fat) ^1^ Whole milk (≈3.5% fat) ^1^ Semi-skimmed milk (≈1.5% fat) ^1^ Skimmed milk (≈0.5% fat) ^1^ I do not drink milk ^1^
	Do you eat the fat that is visible on the meat in your food (lard, pork fat)?	Never (1) Rarely (0.67) Often (0.33) Always (0) I do not eat pork (0.5)
**Salt**	How often do you add salt to your food at the table?	Never (1) Usually, when the food does not taste salty enough (0.5) Sometimes, before tasting (0)
**Sugar**	How often do you drink sweet (sugary) soft drinks?	Never (1) Once a week or less (0.67) A few times a week (0.33) Once a day or more (0)
	How often do you eat sweets?	Never (1) Once a week or less (0.67) A few times a week (0.33) Once a day or more (0)
	How many teaspoons of fine sugar do you use when drinking one cup of coffee or tea? How many cups of coffee/tea do you drink per day?	Space was provided to write a number, which was then multiplied by the number of cups of coffee/tea per day. Range 0–48. The total number was scaled by dividing by 10, and adding 1. Those who scored below −1 were winsorized to −1. The final score ranged from −1 to +1.

^1^ For dairy fats (from milk) and spreads, three measures were calculated for saturated fatty acids (SFA), monounsaturated fatty acids (MUFA), and polyunsaturated fatty acids (PUFA). SFA = the proportion of fats in milk/spreads × 0.714; MUFA = milk/spreads fats × 0.257; PUFA = milk/spreads × 0.029. Score for healthy diet index: SFA/−0.305 + MUFA/−0.305 + PUFA/+ 0.305.

**Table 2:** Sociodemographic and clinical characteristics of participants included in the analyses.

Characteristics	*n*	Mean (SD) or *n* (%)
Baseline age (years)	535	56.4 (3.8)
Age at first follow-up (years)	535	70.4 (3.5)
Age at second follow-up (years)	194	78.1 (3.2)
Follow-up from midlife to first examination (years)	341	13.8 (2.5)
Follow-up from midlife to second examination (years)	194	22.1 (2.5)
Sex	535	
Women		327 (61.1%)
Education (years)	515	8.7 (3.4)
APOE ε4 allele	526	
Carrier		166 (31.6%)
Healthy dietary change during the past year (yes)		
Have modified the quality of fats	535	128 (23.9%)
Have increased the consumption of vegetables	535	286 (53.5%)
Have decreased the use of sugar	535	213 (39.8%)
Have decreased the use of salt	535	159 (29.7%)
Total healthy dietary changes (range 4–8)	535	5.47 (1.3)
Healthy diet index (range 1.7–20.9)	458	6.3 (2.8)
Midlife cardio/cerebrovascular or respiratory conditions	535	
Yes		55 (10.3%)
BMI (kg/m^2^)	535	27.0 (3.7)
Physical activity range (0–5)	511	2.8 (1.4)

**Table 3:** The associations between midlife healthy dietary changes and late-life dementia.

	Model 1	Model 2	Model 3
	OR (95% CI)	OR (95% CI)	OR (95% CI)
Total midlife healthy dietary changes	**0.52 (0.29–0.93)**	**0.53 (0.30–0.95)**	**0.41 (0.20–0.85)**
Modify quality of fats	0.45 (0.10–2.04)	0.48 (0.10–2.17)	0.34 (0.06–1.92)
Increase vegetables	0.76 (0.29–1.94)	0.81 (0.30–2.14)	0.98 (0.31–3.12)
Decrease sugar	**0.36 (0.13–0.97)**	**0.37 (0.14–0.99)**	**0.41 (0.14–1.26)**
Decrease salt	0.57 (0.21–1.55)	0.61 (0.22–1.67)	0.39 (0.12–1.35)

Results from logistic regressions presented as odds ratios (OR), Model 1: age, sex, education, cohort, Model 2: Model 1 + healthy diet index, Model 3: Model 2 + cardio/cerebrovascular or respiratory conditions, BMI, APOE ε4, physical activity. Results with *p* < 0.05 are presented in bold.
